# S01-4: Implementing the systems-based approach to physical activity in Scottish Local Authorities

**DOI:** 10.1093/eurpub/ckae114.200

**Published:** 2024-09-26

**Authors:** Dawn McAuley

**Affiliations:** Sports Scotland

## Abstract

**Purpose:**

To share learning from the application of a pragmatic, evidence-driven, systems-based approach to physical activity policy in Scotland at a national and local level from a sportscotland (national agency for sport) perspective.

**Development:**

sportscotland invests in and work with all 32 local authorities and leisure trusts to deliver shared local and national outcomes and priorities for sport and physical activity.

Working in partnership, sportscotland and Public Health Scotland are supporting local government partners apply the systems-based approach used nationally, to guide the development of local and regional evidence-based physical activity strategies and action plans.

**Implementation and dissemination:**

Local government in Scotland is made up of 32 local authorities which vary in size, demographics, and population. We are implementing the approach across a range of settings including Island, rural, city, and mixed urban/rural.

Collectively we provide leadership and support to enable local partners at a local level to adopt a systems-based approach to physical activity to shape the local strategic direction of physical activity and sport.

We provide support, guidance, and resources to local governance groups to help design and shape the local approach and co-facilitate workshops to engage key stakeholders from health, social care and, local government departments including sport and leisure, education, planning, place and transport.

**Evaluation:**

Each local strategy and associated action plans identify local outcomes, outputs and indicators and local governance groups oversee implementation. Accountability and reporting arrangements vary and are aligned to local structures which include Community Planning Partnership.s, Health Boards and/or Council Committees.

**Conclusions and practical implications:**

Local implementation of the systems-based approach is critical to ensuring we reduce physical inactivity across Scotland.

Successful application of the approach is dependent on local leadership, capacity, and commitment.

Partners provide staff time within existing resources. Financial resources are limited across local government and health, the approach aims to maximise use of existing capital, revenue and human resource.

